# Disparities in food access around homes and schools for New York City children

**DOI:** 10.1371/journal.pone.0217341

**Published:** 2019-06-12

**Authors:** Brian Elbel, Kosuke Tamura, Zachary T. McDermott, Dustin T. Duncan, Jessica K. Athens, Erilia Wu, Tod Mijanovich, Amy Ellen Schwartz

**Affiliations:** 1 Department of Population Health, New York University School of Medicine, New York, NY, United States of America; 2 Wagner Graduate School of Public Service, New York University, New York, NY, United States of America; 3 Cardiovascular Branch, National Heart, Lung, and Blood Institute, National Institutes of Health, Bethesda, MD, United States of America; 4 College of Global Public Health, New York University, New York, NY, United States of America; 5 Department of Humanities and Social Sciences, New York University Steinhardt School of Culture, Education, and Human Development, New York, NY, United States of America; 6 Center for Policy Research, Maxwell School, Syracuse University, Syracuse, NY, United States of America; New York City Department of Health and Mental Hygiene, UNITED STATES

## Abstract

Demographic and income disparities may impact food accessibility. Research has not yet well documented the precise location of healthy and unhealthy food resources around children’s homes and schools. The objective of this study was to examine the food environment around homes and schools for all public school children, stratified by race/ethnicity and poverty status. This cross-sectional study linked data on the exact home and school addresses of a population-based sample of public school children in New York City from 2013 to all corner stores, supermarkets, fast-food restaurants, and wait-service restaurants. Two measures were created around these addresses for all children: 1) distance to the nearest outlet, and 2) count of outlets within 0.25 miles. The total analytic sample included 789,520 K-12 graders. The average age was 11.78 years (SD ± 4.0 years). Black, Hispanic, and Asian students live and attend schools closer to nearly all food outlet types than White students, regardless of poverty status. Among not low-income students, Black, Hispanic, and Asian students were closer from home and school to corner stores and supermarkets, and had more supermarkets around school than White students. The context in which children live matters, and more nuanced data is important for development of appropriate solutions for childhood obesity. Future research should examine disparities in the food environment in other geographies and by other demographic characteristics, and then link these differences to health outcomes like body mass index. These findings can be used to better understand disparities in food access and to help design policies intended to promote healthy eating among children.

## Introduction

The epidemic of childhood obesity poses a public health crisis in the United States (U.S.)[1]. About one in five U.S. children is obese[2]. Racial disparities continue, with Black and Hispanic youth having a greater obesity prevalence than non-Hispanic White and Asian children[2,3]. The causes of the epidemic of childhood obesity are thought to be multifaceted, and include individual, social, and environmental factors.

Though studies of the relationship between the food environment and children’s weight outcomes have found mixed results[4,5], neighborhood disparities in food access may play a key role in driving disparities in weight gain among children[6,7]. If so, neighborhood-based approaches to reducing disparities in food access may be effective in addressing the obesity epidemic among children[8]. Indeed, policies seeking to limit or regulate the number of fast food stores in low-income areas or around schools in an attempt to curb obesity have already been considered or passed[9,10].

Particularly important factors to pay attention to when understanding child-level differences in the food environment are both income and race/ethnicity. Income has consistently been found in previously literature to be associated with obesity rates and is a key health determinant overall. Race/ethnicity is similarly a variable highly associated with obesity[11]. Important for both of these variables, they are associated with key differences in where children live, they go to school, and therefore their subsequent food environment[12]. Finally, there are historical and current disadvantages among key groups, namely low-income and racial/ethnic minority groups, which make understanding such differences much more critical, and do so in a way that does not merely explain them away[13].

Previous studies documenting children’s food access by race/ethnicity and income suggest important disparities exist, but limitations in data and methods indicate additional work is warranted[14–16]. Most studies are limited by: a) relying upon administrative boundaries (e.g. zip codes) that may poorly represent where the child lives or goes to school to define access[7]; b) considering food outlet types in isolation (e.g., just fast-food, but not supermarkets); c) evaluating food access near home or school, but not both; d) operationalizing food access with a single measure (e.g., distance to nearest or density but not both); or e) all or many of the above[7,17].

The US Department of Agriculture (USDA) reports that just over 6% of the total population resides further than one mile from a supermarket and lives in “low-income and low-supermarket-access tracts”[18]. Food access disparities by income are well established in the research literature, along with disparities by race/ethnicity[19]. A 2016 study by D’Angelo et al. on socioeconomic and demographic disparities in fast-food access around U.S. schools in 97 counties across 40 states suggests that over 50% of schools with predominantly Hispanic students, and students from low income families (not including Black students), had fast-food outlets nearby as compared to only 21% of schools with predominantly White students[20]. Other studies using national datasets present similar findings, but go a step further to look at disparities by region. For example, Bower et al. (2014) showed that neighborhoods with greater poverty have lower availability of supermarkets (regardless of race/ethnicity). Furthermore, predominantly black census tracts had the least amount of supermarkets while white tracts had the most. However, no associations between poverty and race and supermarket access were evident in rural areas[21]. Similarly, Grimm et al. (2013) found that income and healthy food access varied by US region (e.g., Northeast, South, Midwest, and West) with no association seen in the West. Collectively, research on food access across the U.S. shows disparities in access to healthy (often considered supermarkets, as described in more detail below) and unhealthy food (often fast food or corners store, per below) are often driven by race/ethnicity and income level, yet differentially exhibited by region or setting [7,22]. While the research on food access and neighborhoods has expanded, much of it–including the cited studies–suffers from one or more of the methodological challenges noted in the previous paragraph, often based on limitations in the available person-level of store level data.

In New York City (NYC), where our study is based, a few prior studies have examined food access[12,23,24]. Kwate et al., for example, assessed density of fast-food restaurants in relation to race and socio-economic status (SES)[12]. They found that primarily Black neighborhoods (defined as a census block group with > 70% Black residents) had a higher density of fast-food outlets compared to primarily White neighborhoods (defined as a census block group with > 70% White residents). In addition, Black neighborhoods had relatively similar fast-food exposure irrespective of income levels. Separate research conducted by Kwate et al. (2010), assessed the proximity of schools to fast-food outlets[23]. They reported that more than half of public schools had at least one fast-food restaurant within 400 meters, while private schools had less availability. They also found that the proportion of Blacks in public elementary and high schools was associated with the number of fast-food restaurants in the immediate neighborhood around the school[23]. A shortcoming of these two studies[12,23] is a focus on only fast-food outlets and an emphasis on census block groups[12] rather than more sensitive measures such as buffers around each student’s home or school.

In another study Neckerman et al. (2010) examined neighborhoods surrounding public schools in NYC in relation to five food outlet types, including national chain fast-food restaurants, local chain fast-food restaurants, convenience stores, pizza shops, and small grocery stores[24]. They described school food neighborhoods by counting food stores within 400- and 800-m buffers around each school. They found that exposure to these types of food outlets around schools was significantly associated with the proportions of ethnic minority and low-income students at each school. A key limitation of this study is the exclusive focus on schools and only less healthy food sources, such as fast food restaurants and corner stores, rather than more healthy food sources such as supermarkets.

The aim of the current study is to address the aforementioned methodological challenges and examine potential disparities in food access around the homes and schools of children in New York City, stratified by race/ethnicity and poverty status. Our goal is not to explain away or predict why such differences occur in this work but simply understand the different experiences of these groups of children. We hypothesize that, based on limited past literature, minority or poor students will have less access to healthy food and greater access to unhealthy food.

## Methods

This study was conducted according to the guidelines laid down in the Declaration of Helsinki and all procedures involving human subjects were approved by the New York University School of Medicine Institutional Review Board; study #S13-00403. The study uses data previously collected as part of students’ education. Students who do not want to participate in the activity, or parents who do not want their children to participate, are able to opt out of participation in the activity through their schools. However there is no consent process as it is a part of education; the NYU School of Medicine's Institutional Review Board approved a waiver of consent.

To address the aforementioned limitations in food access research, we examined data on essentially all public school students in NYC by geocoding students’ home and school addresses, which allowed us to measure food outlets around both locations. Approximately 83% of all school aged children in NYC are enrolled in public school[25]. Additionally, we assessed multiple food outlet types for each student and by race/ethnicity and poverty status, including corner stores, fast-food restaurants, wait-service restaurants, and supermarkets. First, we examined the mean distance to the nearest food outlet of all types from each student’s home and then from each student’s school. Second, we investigated the mean count of food facilities within a 0.25 mile buffer around their homes and schools. We choose this measure because it is about a five minute walk for most people[26]. Third, we use alternative measures with 0.1 mile and 0.5 mile as a robustness check. We decided to report the 0.25 mile buffer as our primary outcome to best capture and characterize the NYC food environment. The 0.1 buffer is only about two city blocks, and likely too granular and thus insufficient as a reference point to most of NYC outside of densely populated Manhattan. Though the 0.5 measure accommodates outer boroughs well, it is an unrealistic representation of the Manhattan food environment. Though there is limited literature available to determine the optimal buffer size when considering the food environment, these small distances are consistent with some prior work examining childhood obesity[27]. For both measures, we examined the differences by poverty status and its interaction with race/ethnicity. We purposely did not control for additional contextual measures, such as population density, since we wanted to understand the absolute differences experienced by these students and not whether they could be explained by or correlated with contextual factors.

### Study participants

Data came from the NYC Department of Education (DOE), and included administrative student-level data on race/ethnicity (i.e., White, Black, Hispanic, and Asian & Other), eligibility for free or reduced price lunch, and students’ geocoded home addresses. We defined students as low-income if we observed that they were eligible for free or reduced-priced lunch at any time between 2001–2013 (defined by family income below 185% of the Federal Poverty line), as poverty is often persistent, and students did not consistently report their eligibility for free or reduced-priced lunch, due to stigma and other reasons. It is noteworthy that at the time of data collection by DOE, students did not yet have the option to report multi-race.

**Fig 1:**
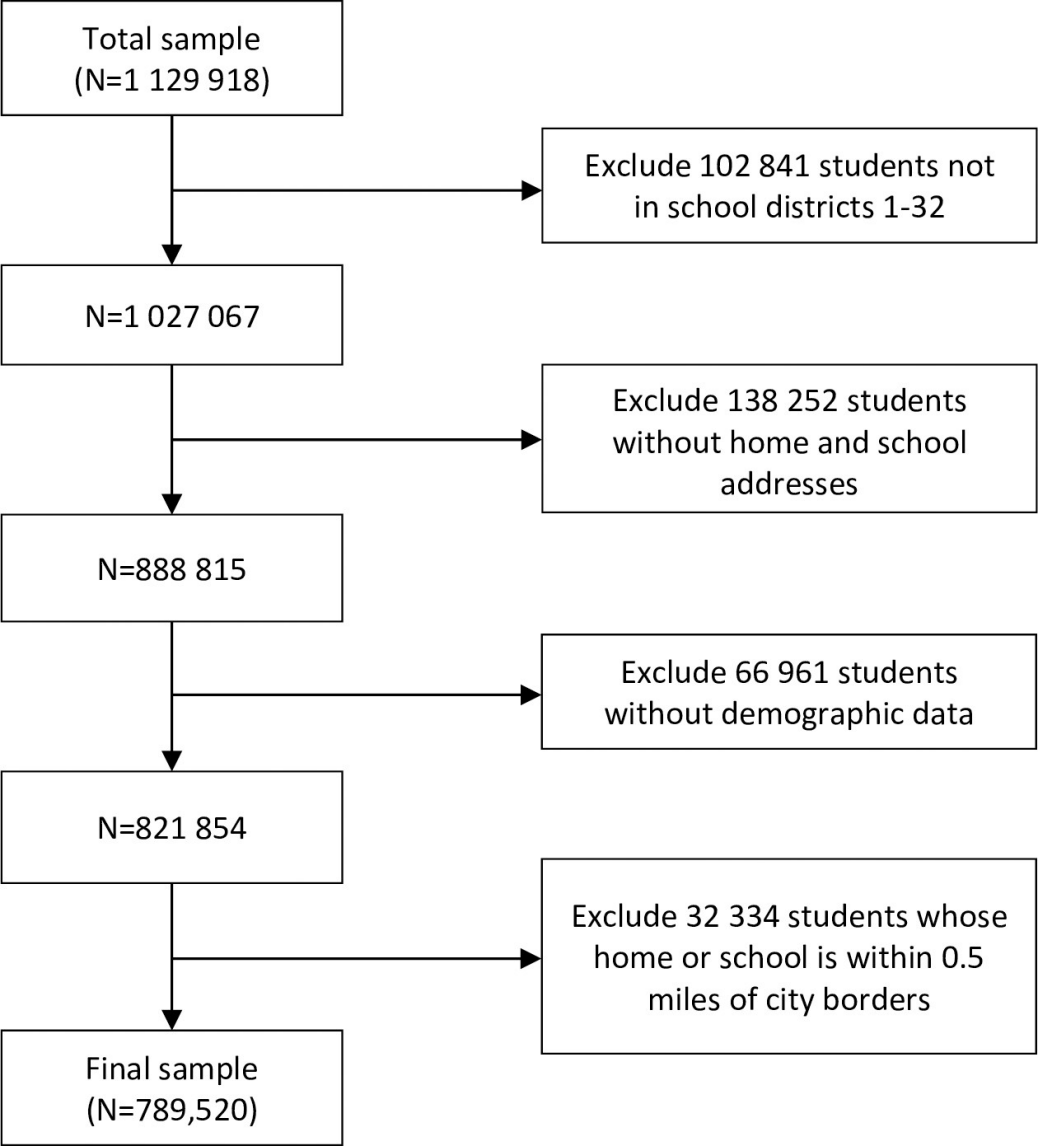
Flow diagram for sample size.

The sample for this study included NYC public school students with home and school addresses, and student-level demographic data (n = 1,129,918), which include grade, race/ethnicity and whether a student qualified for free or reduced-priced lunch. We excluded students that: a) were enrolled in charter schools, special education only schools, pre-K, and juvenile detention centers (n = 102,841) because the quality of the demographic data received from these schools was poor, b) did not have home and school addresses (n = 138,252), c) lacked demographic data (n = 66,961), or d) lived within 0.5 miles of the Westchester or Nassau county borders (i.e. those living in the northernmost part of the Bronx and easternmost part of Queens), where we could not capture their complete food environment (n = 32,334). These exclusions resulted in a total analytic sample of 789,520 students in 2013. See Fig 1 for a flow diagram illustrating how we arrived at our final sample size.

### Food environment measures around students’ home and school

We created four food outlet variables, which together account for 93% of all non-mobile food outlets in NYC: 1) corner stores (bodegas and smaller groceries), defined here as stores less than 6,000 square feet in accordance with NYC’s Food Retail Expansion to Support Health Program (FRESH) initiative (these account for 90.8% of all non-restaurant food stores in NYC)[28], 2) fast-food restaurants (national chain and non-chain outlets specified as “fast food” and without wait service indicated; 60.1% of all restaurants)[29,30], 3) wait-service restaurants (national chain and non-chain outlets that include any type of wait service indication; 39.9% of all restaurants), and 4) supermarkets (food stores greater than 6,000 square feet; 9.2% of all non-restaurant food outlets). Locations of all restaurants were from the NYC Department of Health and Mental Hygiene Restaurant Grading data, which include restaurant inspections that occur at least annually. Locations of all other food outlets were from the New York State Department of Agriculture and Markets Licensing and Inspection data, which include information from location visits that occur approximately every 18 months, though inspections may be more or less frequent. Consistent with previous literature, we generally consider corner stores to be less healthy food outlets because they do not often have fresh food available[31], and supermarkets to be more healthy food outlets[32] because they do. We consider fast food restaurants to be less healthy as they often offer calorie dense food, and wait-service restaurants more so as there are relatively a wider range of options[33]. Previous literature has also shown that even though “healthy” food outlets could offer unhealthy options, and vice versa, it is common that costumers more often purchase calorie dense foods at corner stores and fast-food restaurants[34].

For each of the food outlet types, we constructed two food proximity measures from each student's home and school: 1) the distance to the nearest outlet, and 2) a count of the number of food outlets within a 0.25 mile buffer, or about 5 blocks and a five minute walk, a distance that is also consistent with prior work measuring the food environment[4,26]; sensitivity analysis was performed, per below, including those that looked at slightly higher and lower walking distances. Distance was calculated in ArcGIS 10.5 (Redlands, CA) using the street network distance between the geographic coordinates of students (homes or schools) and the food outlets. Geocoding was done using the NYC Department of City Planning’s Geosupport Desktop Edition software (New York, NY), which is optimized to handle NYC-specific addresses.

### Statistical analysis

We examined disparities in access to each food outlet type (i.e., nearest distance and count) for 789,520 public school students in NYC in Academic Year 2013. First, we summarized the students’ characteristics. For each measure we presented and plotted the mean distance to the nearest food outlet and the number of food outlets for each race/ethnicity and poverty subgroup. We performed pair-wise T-tests between each of these subgroups (with interaction) and every other result on the same figure, using the Bonferroni correction for multiple comparisons (p-values reported in Results take into account the correction). We also performed joint F-tests of home and school measurements (for school, we first ran a regression with the school measurement as the outcome, and the interaction terms as the independent variables with clustered standard errors at school level. We then tested the statistical significance on the coefficients with the *test* command in Stata). As a sensitivity analysis, we looked at results separated by grade (K-5, 6–8, and 9–12). For all analyses we used Stata version 15 (College Station, TX).

## Results

Students’ socio-demographic characteristics are shown in Table 1. Students were predominantly Hispanic (41%) and Black (26%), followed by Asian & Other (17%) and White (16%). The majority of the students (85%) were classified as low-income. The mean age of the students was 11.8 years (SD ± 4.0 years). Most students resided in Queens (32%), followed by Brooklyn (29%), the Bronx (22%), Manhattan (11%), and Staten Island (6%).

**Table 1:** Students' socio-demographic characteristics, 2013.

		Race/ethnicity				Poverty status	
	Total N = 789520	White n = 123 874	Black n = 205 875	Hispanic n = 327 220	Asian & Other n = 132 551	Low-income n = 669 478	Not low-income n = 120 042
Female, %	50	49	51	50	49	50	49
Low-income, %	85	55	93	93	80	100	0
Foreign born, %	16	13	11	15	31	17	11
Special education, %	13	13	15	16	5	14	10
English at home, %	56	69	92	40	28	53	72
Below proficient score on NYSESLAT^a^, %	14	7	3	22	21	15	9
Grade, mean (SD)	5.97 (3.75)	5.59 (3.74)	6.37 (3.71)	5.84 (3.74)	5.99 (3.79)	6.24 (3.70)	4.42 (3.63)
Age, mean (SD)	11.78 (3.97)	11.27 (3.86)	12.28 (3.95)	11.69 (3.98)	11.72 (3.98)	12.09 (3.93)	10.05 (3.74)
Borough, %							
Manhattan	11	14	8	14	8	10	18
Bronx	22	5	23	34	5	24	8
Queens	32	34	48	23	31	33	30
Brooklyn	29	26	19	27	52	28	31
Staten Island	6	21	3	3	3	5	13

**Notes:** Sample includes NYC public school students in districts 1–32 with home and school address data and student-level demographic data. Students for whom a substantial proportion of their food environment lies outside of the city boundaries (those whose home or school is within half a mile from city borders) are excluded.

^a^ NYSESLAT stands for the New York State English as a Second Language Achievement Test.

**Fig 2:**
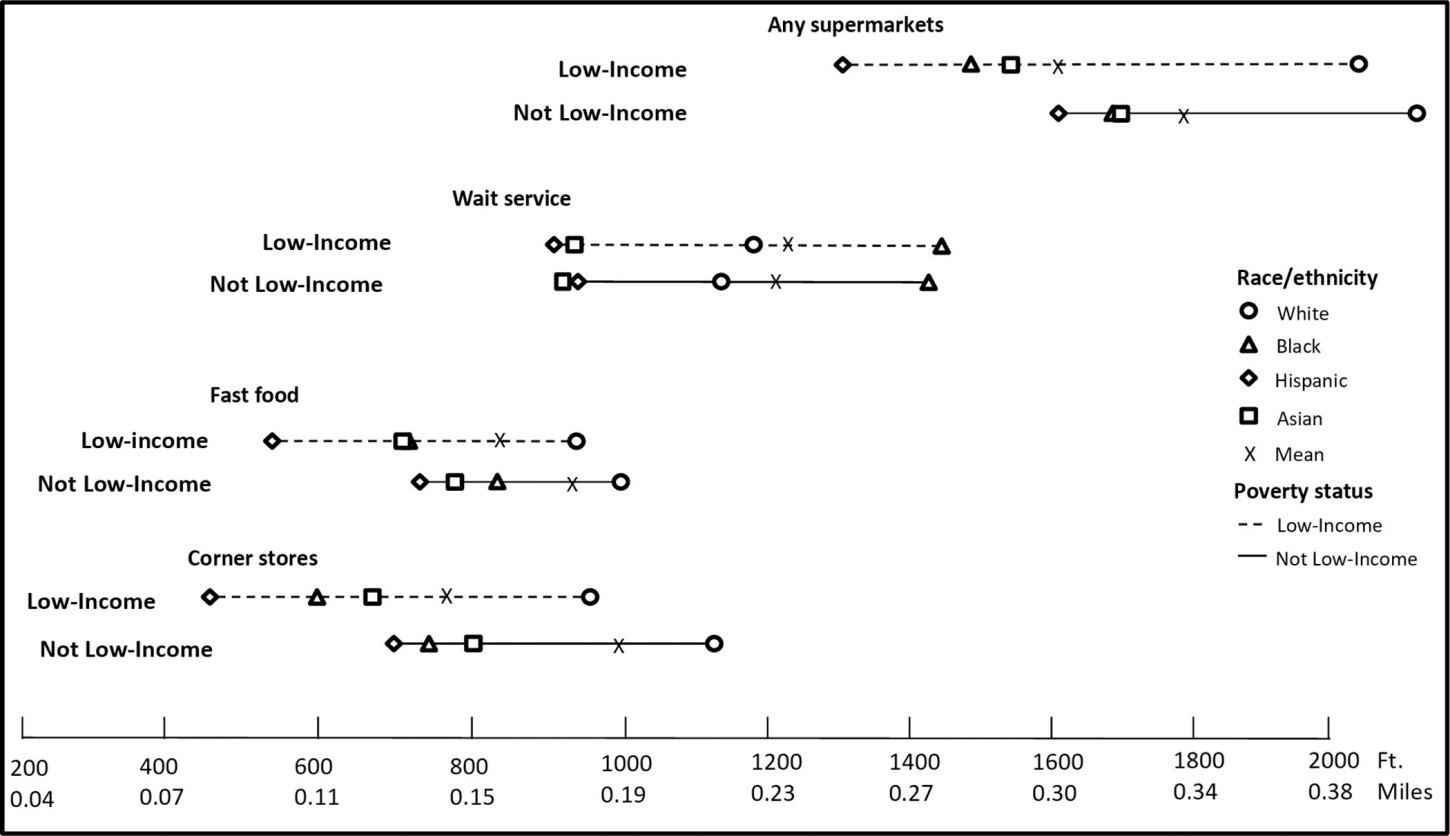
Mean nearest distance in feet to food facilities from home by race and poverty interactions in 2013. We conducted a joint F-test, which suggests significant differences among income and race groups (p<0.05), and pair-wise T-tests for multiple comparisons based on Bonferroni correction (28 pairs in total, with each pair tested on all four food outlets, separately). The majority of comparisons were statistically significant (p<0.05). S18 Table presents test results (p-values) for each pair.

**Fig 3:**
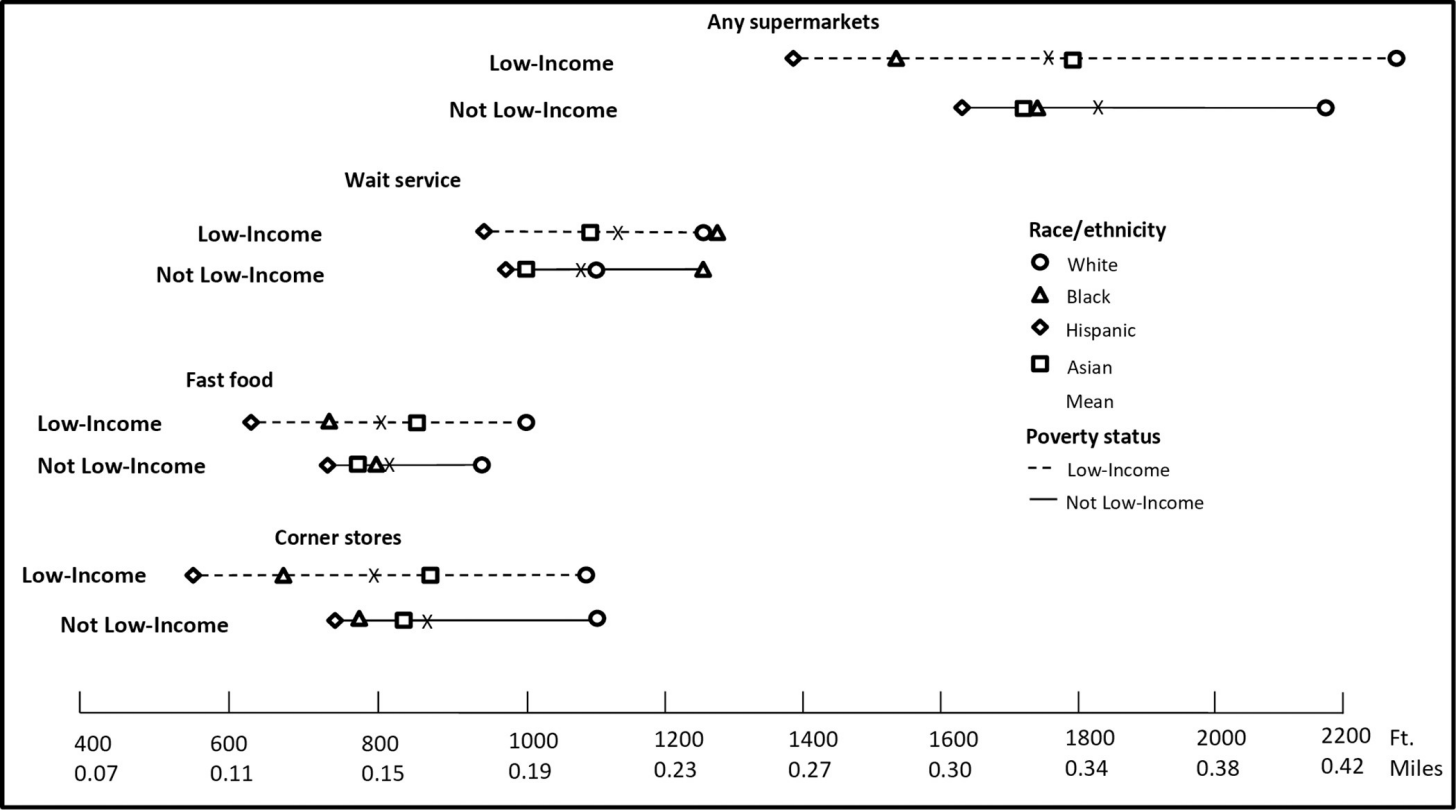
Mean nearest distance in feet to food facilities from school by race and poverty interactions in 2013. We conducted joint F-test with school level clustered errors suggests significant differences among income and race groups (p<0.05). And pair-wise T-tests for multiple comparisons based on Bonferroni correction (28 pairs in total, with each pair tested on all four food outlets, separately). More often than not, the T-test indicated no statistically significant difference. S19 Table presents test results (p-values) for each pair.

The mean distances to the nearest food outlets of all types from homes and schools by race/ethnicity and poverty interactions are presented in Figs 2 and 3 (S1 Table gives the exact data points in table format). Overall, the closest distance to each type of food outlet from homes by poverty status indicates similar patterns across race/ethnicity. For example, not low-income Hispanic students lived closer to corner stores by 440 feet, fast-food restaurants by 277 feet, wait-service restaurants by 179 feet and supermarkets by 562 feet compared to not low-income White students (all p<0.05). For both low-income and non-low-income children, Black, Hispanic, and Asian students lived and attended schools closer to nearly all food outlet types, compared to White students (p<0.05), except that Black students lived farther from wait-service outlets around both homes and schools, compared to White students. For each food outlet type, we compared every race/income subgroup of students against each other, and the majority were statistically significantly different, (p<0.05, including the Bonferroni correction, see S1 Table notes for exceptions).

**Fig 4:**
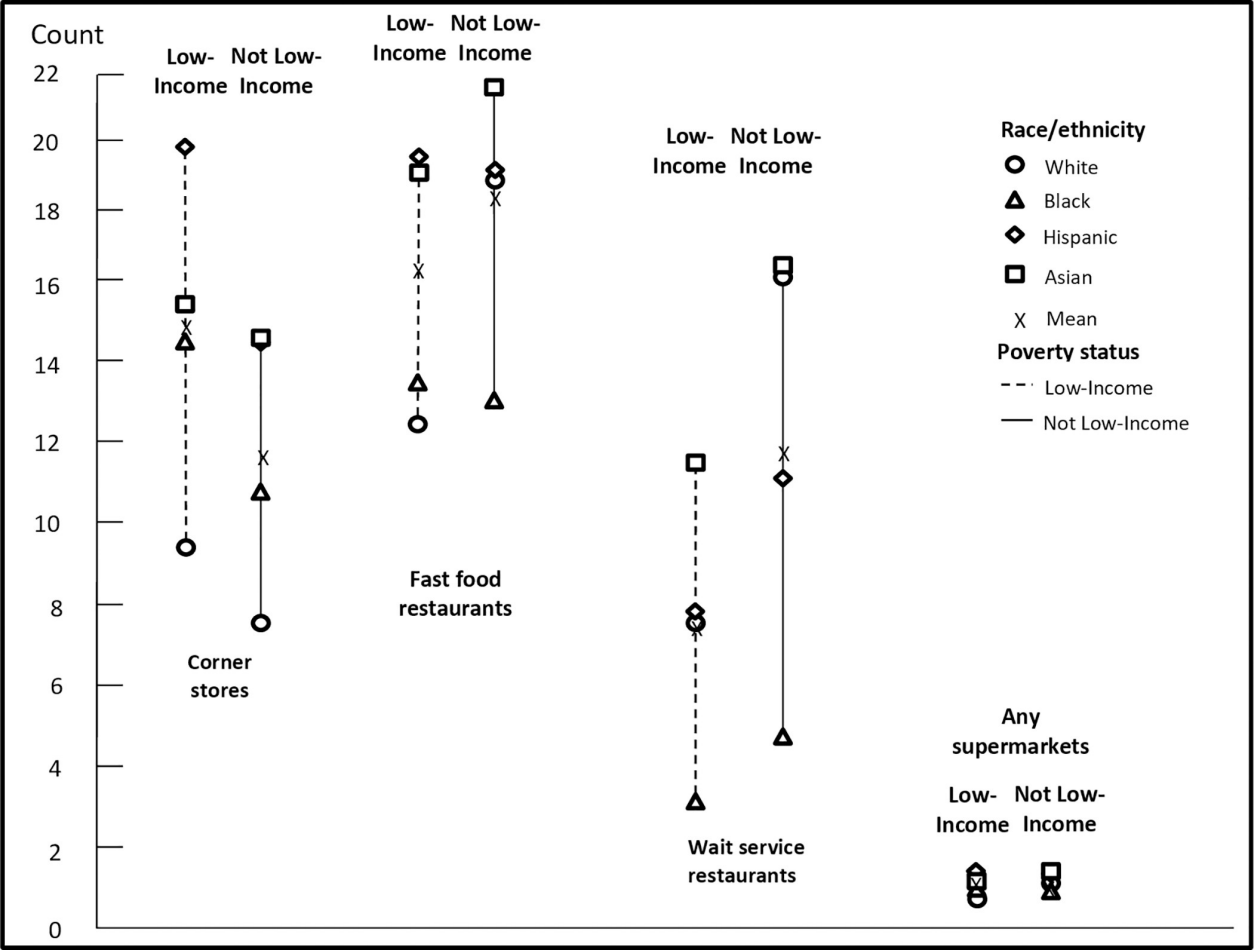
Mean count within 0.25 miles (1,320 ft) of food facilities from home, race and poverty interactions, 2013. We conducted a joint F-test, which suggests significant differences among income and race groups (p<0.05), and pair-wise T-tests for multiple comparisons based on Bonferroni correction (28 pairs in total, with each pair tested on all four food outlets, separately). The majority of comparisons were statistically significant (p<0.05). S20 Table presents test results (p-values) for each pair.

**Fig 5:**
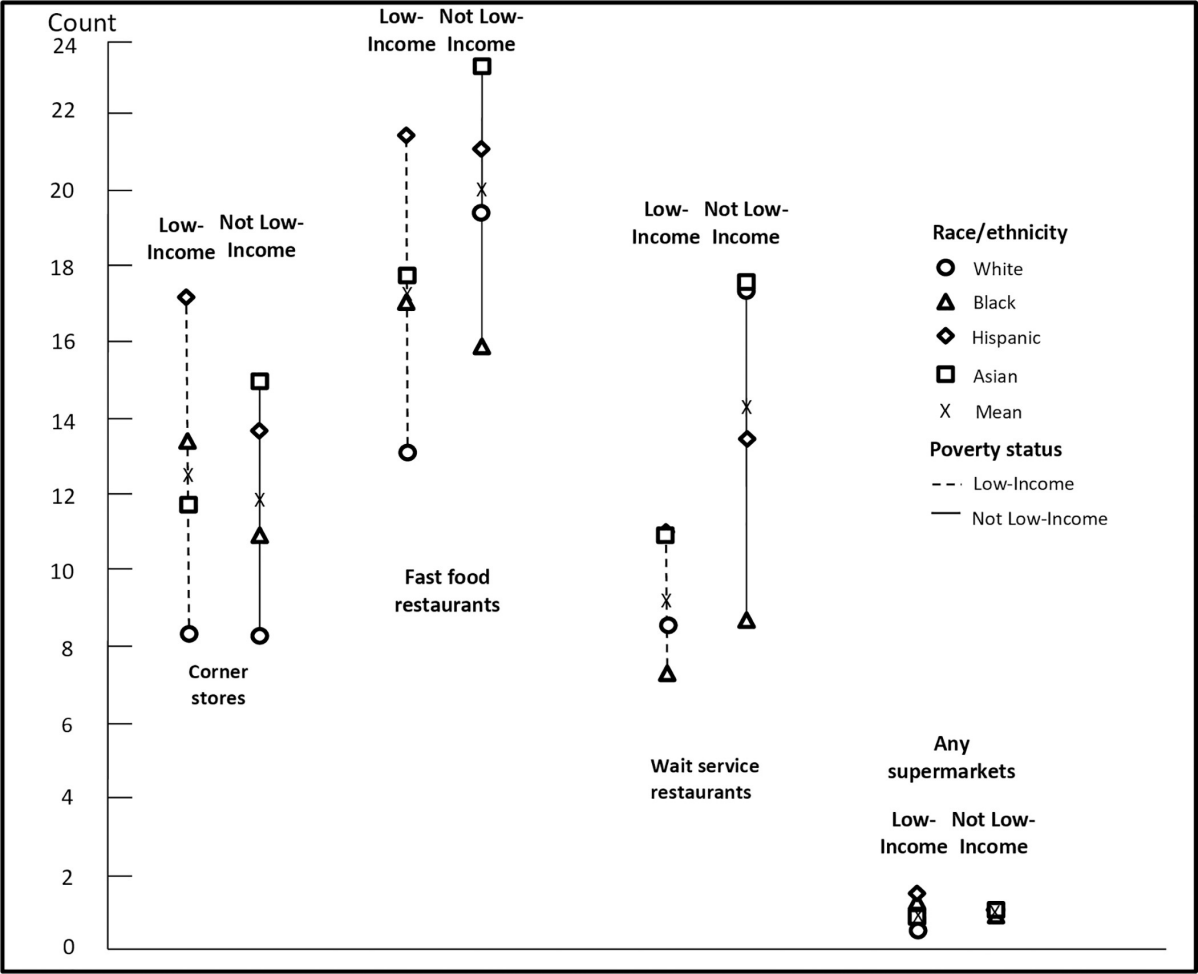
Mean count within 0.25 miles (1,320 ft) of food facilities from school, race and poverty interactions, 2013. We conducted joint F-test with school level clustered errors suggests significant differences among income and race groups (p<0.05). And pair-wise T-tests for multiple comparisons based on Bonferroni correction (28 pairs in total, with each pair tested on all four food outlets, separately). More often than not, the T-test indicated no statistically significant difference. S21 Table presents test results (p-values) for each pair.

The mean count of food outlets within 0.25 miles (1320 ft) of homes and schools by race/ethnicity and poverty interactions is shown in Figs 4 and 5 (S2 Table). Overall, among not low-income students, Hispanic and Asian students had greater access from home to corner stores and supermarkets than White students, and Black students had greater access from home to corner stores than White students. For example, not low-income Black and Hispanic students had access to 2.4 and 5.5 more corner stores, respectively, compared to not low-income White students (p<0.05). Low-income Black, Hispanic, and Asian students had access to 4.7, 10.4, and 5.9 more corner stores; 1.0, 7.2, and 6.6 more fast-food restaurants; and 0.3, 0.6, and 0.4 more supermarkets from home, respectively, compared to low-income White students (all p<0.05). Patterns for mean counts of food outlets within 0.25 miles of schools were similar across race/ethnicity and poverty status interactions, in that low-income Black, Hispanic, and Asian students had greater access from school to corner stores compared to low-income White students. As we did for distance measures, all counts for each food type were compared and the vast majority of comparisons were statistically significant (p<0.05, including the Bonferroni correction).

In a sensitivity analysis, results were robust to alternative buffer sizes (i.e., 0.1 miles and 0.5 miles; see S3 and S4 Tables). They were also robust to analyses by student grade (i.e., K-5, 6–8, 9–12; see S5–S16 Tables). Some differences in magnitude do exist, but nothing that changes the ordering of results, except in the case of not low-income students in grades 9–12 where results for fast-food outlets and supermarkets tend to equalize. Additionally, we also compared mean and median measurements on the distance to the nearest food outlets of all types and the count of food outlets within 0.25 miles (S17 Table). Data suggest that for wait service restaurants (count measure), a fair amount of students are somewhat skewed towards the lower end (having 0–2 such restaurants within 0.25 miles of their homes). However, aside from this, the mean and median measurements yield similar results, particularly in terms of ordering and relative magnitude, and examining median does not qualitatively change our interpretation. Finally, our main goal was to describe the differences among children of difference race/ethnicity and income levels instead of explaining what drives these differences. That said, we also examined results that clustered the standard errors at the school level (see S1 and S2 Tables). In doing so, we find that the F-test for joint significance of race/income is significant and many of the main effects are as well, even though not all individual differences remain significantly different (given the often segregated nature of NYC public schools).

## Discussion

In our study of nearly 800,000 public school students in NYC, we examined disparities in food access by calculating the nearest distance to and the count of food outlet types, including corner stores, fast-food, wait-service, and supermarkets within 0.25 miles around the students’ home and school addresses. Overall, we found that Black, Hispanic, and Asian students live and attend schools closer to nearly all food outlets (corner stores, fast food outlets, wait service restaurants, and supermarkets) compared with White students, regardless of poverty status. Among not low-income students, Black, Hispanic, and Asian students were closer from home and school to corner stores and supermarkets, and had more supermarkets around their schools than White students. The majority of mean nearest distances to and counts of food outlets by type differed by poverty status across race/ethnicity.

Our findings are consistent with and build upon previous work examining children’s food environments, which generally focuses on the density of fast-food outlets around schools. A recent study examining food access in terms of distance and density around schools in Boston found that higher income neighborhoods had lower densities of fast-food restaurants and were farther from fast-food restaurants and convenience stores compared with lower income neighborhoods[35]. Another study examined density of fast-food outlets around schools in Chicago and found that fast-food restaurants were clustered around these locations[36]. A third study examining the proximity of fast-food outlets to schools in Los Angeles County reported that they were more likely to be located around schools in low-income neighborhoods compared to schools in the high-income neighborhoods[37]. Finally, one study examined disparities in food environments around schools and reported that Hispanic students tended to attend schools with greater access to convenience stores and restaurants[38].

Our work is also consistent with studies examining food access among children around schools[20,23,24] and fast-food density specifically in NYC neighborhoods[12]. These studies found that students who are from non-White racial/ethnic groups and low-income families tended to attend schools near unhealthy food outlets[20,23,24]. This finding is supported with our study; Black, Hispanic, and Asian students overall had greater access to fast-food and corner stores than White students. Notably, however, our work finds that Black, Hispanic, and Asian students’ access to all food outlets was greater than their White counterparts. This meant that they also, unexpectedly, had greater access to healthier food outlets, particularly supermarkets. The findings from our study underscore the complexity of disparities in food access across various types food outlets in NYC, resulting in tremendous access to both healthy and unhealthy food outlet types by non-White and low-income students.

Our findings also suggest poverty is important, as we found that low-income students live closer to nearly all types of food outlets than not low-income students. This finding supported in a recent study that examined the density of fast-food outlets at the census-block group level across NYC[12]. The researchers found that predominantly Black neighborhoods had greater fast-food density compared to predominantly White neighborhoods. High- and low-income Black neighborhoods had almost the same exposure level to fast-food outlets. This finding suggests that racial segregation could play a role in food access in NYC[12], as well as in other major cities in the U.S.[39,40].

This study has a number of strengths. It is the first study of which we are aware to examine food access around both the home and school environments using student-level address data. As well, it is the first study of which we are aware to examine a wide range of food store types and assess how race/ethnicity and poverty status interact in relation to food access disparities. Additionally, we examine both the nearest distance to and density of food outlets, allowing for a full view of the food landscape[35].

However, there are some noteworthy limitations. First, despite having the population of students from the largest school system in the country, a significant advancement on prior literature, these children are predominantly low-income and non-white (at least compared to national data). We also do not have data on the 17% of children who are in private or charter schools, who are more likely than public school children in NYC to be white and higher income with lower subsequent obesity rates[41,42]. As such, our data may not be generalizable to all students, in NYC or otherwise. Our sample is 15.7% white (representing 123,874 kids), compared with 25.3% city-wide during the same period. That said, we still have a very broad sample, with enough representation amongst all our key groups to allow for comparisons. Relatedly, some boroughs within New York City, such as Manhattan, are different than other urban and non-urban locations. Differences in density and access to private automobiles are two considerations. Concepts such as “food deserts” are often not used to describe NYC, where access is measured differently. As such, our results may not be generalizable to other locations.

Second, we lack data on small mobile food carts and sidewalk stands. We also did not have data on whether particular stores sold more or less healthy products, and largely relied on proxies like size or service type to determine whether an outlet type should be categorized as healthy or unhealthy. Such categorization also does not take into consideration the cost or quality of food items sold at these outlets. It is worth noting, for example, that unhealthy foods typically sold in corner stores are also sold at supermarkets, and usually in great variety and often at lower prices. Similarly, there are now fast food outlets selling traditionally healthier foods, such as salads. Third, given the goals of determine true differences amongst children, the study does not examine what drives differences among groups, and this could be the focus of future work. Fourth, there is no consensus in the literature on the most meaningful buffer size to consider around home and school, though our sensitivity analyses of different buffer sizes (S3 and S4 Tables) did not change the results. Like administrative boundaries, we recognize that buffer-based neighborhood definitions suffer from their own limitations[17]. One limitation of buffer-based neighborhood definitions is they make isotropic assumptions regarding one’s exposure to specific neighborhood environments. Relatedly, this work suffers from the “uncertain geographic context problem”[43] as we do not know how much time children spend in their home and school neighborhoods. Additionally, the cross-sectional nature of the data we used limits conclusions we can make about changes in the food environment over time, something that is pertinent in future research. Finally, some students may be misclassified as not low-income, as it is possible that some students did not submit forms for free or reduced-priced lunch due to stigma and other issues.

## Conclusions

We examined food access around homes and schools among a large sample of public school students in NYC, by race/ethnicity and poverty status interactions. Our findings unexpectedly show that among this sample there is significant access to all types of food outlets, not just unhealthy food outlets as hypothesized. Black, Hispanic, and Asian students, regardless of poverty status, had greater access to corner stores, fast-food restaurants, and supermarkets around homes and schools than did White students. Our findings can be used to better understand the disparities in food access and to design policies promoting healthy eating among children. In particular, we believe these findings demonstrate a need to develop policies that are not focused predominantly or exclusively on access. Rather than just working to improve access to stores like supermarkets—which are already available and abundant around children’s homes and schools—more attention could be given to improving other aspects of the food environment, such as the quality and cost of food that is available in these existing food outlets. This study establishes a base through which we can consider additional research and policy and program implications; in describing the existing disparities, we can better validate how such disparities come about, shifting the focus of policies and programs from equality in access to equity in access. Future research should examine disparities in the food environment in other geographies and by other demographic characteristics, and then link these differences to health outcomes like body mass index (BMI).

## Supporting information

S1 Table Mean nearest distance (in ft.) to food facilities from home and school, race and poverty interactions, AY2013. Sample includes NYC public school students in districts 1–32 with home and school address data and student-level demographic data. Students for whom a substantial proportion of their food environment lies outside of the city boundaries (those whose home or school is within half a mile from city borders) are excluded. For both home and school measurements, we conducted joint F-tests, which suggest significant differences (p<0.05), and pair-wise T-tests for multiple comparisons based on Bonferroni correction (28 pairs in total, with each pair tested on all four food outlets, separately). The majority of comparisons for home measurements were statistically significant (p<0.05) while for the school measurements, more often than not, the results indicated no statistically significant difference. S18 and S19 Tables present the T-test results (p-values), respectively.
(PDF)

S2 Table Mean count within 0.25 miles of food facilities from home and school, race and poverty interactions, AY2013. Sample includes NYC public school students in districts 1–32 with home and school address data and student-level demographic data. Students for whom a substantial proportion of their food environment lies outside of the city boundaries (those whose home or school is within half a mile from city borders) are excluded. For both home and school measurements, we conducted joint F-tests, which suggest significant differences (p<0.05), and pair-wise T-tests for multiple comparisons based on Bonferroni correction (28 pairs in total, with each pair tested on all four food outlets, separately). The majority of comparisons for home measurements were statistically significant (p<0.05) while for the school measurements, more often than not, the results indicated no statistically significant difference. S18 and S19 Tables present the T-test results (p-values), respectively.
(PDF)

S3 Table Mean count within 0.10 miles of food facilities from home and school, race and poverty interactions, AY2013. Sample includes NYC public school students in districts 1–32 with home and school address data and student-level demographic data. Students for whom a substantial proportion of their food environment lies outside of the city boundaries (those whose home or school is within half a mile from city border are excluded.
(PDF)

S4 Table Mean count within 0.50 miles of food facilities from home and school, race and poverty interactions, AY2013. Sample includes NYC public school students in districts 1–32 with home and school address data and student-level demographic data. Students for whom a substantial proportion of their food environment lies outside of the city boundaries (those whose home or school is within half a mile from city borders) are excluded.
(PDF)

S5 Table Mean nearest distance (in ft.) to food facilities from home and school, race and poverty interactions, Grade K-5, AY2013. Sample includes NYC public school K-5 students in districts 1–32 with home and school address data and student-level demographic data. Students for whom a substantial proportion of their food environment lies outside of the city boundaries (those whose home or school is within half a mile from city borders) are excluded.
(PDF)

S6 Table Mean count within 0.25 miles of food facilities from home and school, race and poverty interactions, Grade K-5, AY2013. Sample includes NYC public school K-5 students in districts 1–32 with home and school address data and student-level demographic data. Students for whom a substantial proportion of their food environment lies outside of the city boundaries (those whose home or school is within half a mile from city borders) are excluded.
(PDF)

S7 Table Mean count within 0.1 miles of food facilities from home and school, race and poverty interactions, Grade K-5, AY2013. Sample includes NYC public school K-5 students in districts 1–32 with home and school address data and student-level demographic data. Students for whom a substantial proportion of their food environment lies outside of the city boundaries (those whose home or school is within half a mile from city borders) are excluded.
(PDF)

S8 Table Mean count within 0.5 miles of food facilities from home and school, race and poverty interactions, Grade K-5, AY2013. Sample includes NYC public school K-5 students in districts 1–32 with home and school address data and student-level demographic data. Students for whom a substantial proportion of their food environment lies outside of the city boundaries (those whose home or school is within half a mile from city borders) are excluded.
(PDF)

S9 Table Mean nearest distance (in ft.) to food facilities from home and school, race and poverty interactions, Grade 6–8, AY2013. Sample includes NYC public school 6–8 grade students in districts 1–32 with home and school address data and student-level demographic data. Students for whom a substantial proportion of their food environment lies outside of the city boundaries (those whose home or school is within half a mile from city borders) are excluded.
(PDF)

S10 Table Mean count within 0.25 miles of food facilities from home and school, race and poverty interactions, Grade 6–8, AY2013. Sample includes NYC public school 6–8 grade students in districts 1–32 with home and school address data and student-level demographic data. Students for whom a substantial proportion of their food environment lies outside of the city boundaries (those whose home or school is within half a mile from city borders) are excluded.
(PDF)

S11 Table Mean count within 0.1 miles of food facilities from home and school, race and poverty interactions, Grade 6–8, AY2013. Sample includes NYC public school 6–8 grade students in districts 1–32 with home and school address data and student-level demographic data. Students for whom a substantial proportion of their food environment lies outside of the city boundaries (those whose home or school is within half a mile from city borders) are excluded.
(PDF)

S12 Table Mean count within 0.5 miles of food facilities from home and school, race and poverty interactions, Grade 6–8, AY2013. Sample includes NYC public school 6–8 grade students in districts 1–32 with home and school address data and student-level demographic data. Students for whom a substantial proportion of their food environment lies outside of the city boundaries (those whose home or school is within half a mile from city borders) are excluded.
(PDF)

S13 Table Mean nearest distance (in ft.) to food facilities from home and school, race and poverty interactions, Grade 9–12, AY2013. Sample includes NYC public school 9–12 grade students in districts 1–32 with home and school address data and student-level demographic data. Students for whom a substantial proportion of their food environment lies outside of the city boundaries (those whose home or school is within half a mile from city borders) are excluded.
(PDF)

S14 Table Mean count within 0.25 miles of food facilities from home and school, race and poverty interactions, Grade 9–12, AY2013. Sample includes NYC public school 9–12 grade students in districts 1–32 with home and school address data and student-level demographic data. Students for whom a substantial proportion of their food environment lies outside of the city boundaries (those whose home or school is within half a mile from city borders) are excluded.
(PDF)

S15 Table Mean count within 0.1 miles of food facilities from home and school, race and poverty interactions, Grade 9–12, AY2013. Sample includes NYC public school 9–12 grade students in districts 1–32 with home and school address data and student-level demographic data. Students for whom a substantial proportion of their food environment lies outside of the city boundaries (those whose home or school is within half a mile from city borders) are excluded.
(PDF)

S16 Table Mean count within 0.5 miles of food facilities from home and school, race and poverty interactions, Grade 9–12, AY2013. Sample includes NYC public school 9–12 grade students in districts 1–32 with home and school address data and student-level demographic data. Students for whom a substantial proportion of their food environment lies outside of the city boundaries (those whose home or school is within half a mile from city borders) are excluded.
(PDF)

S17 Table Comparison of Mean and Median Measurements on Distance to Nearest Food Outlet and Count of Outlets within 0.25 Miles, AY2013. (PDF)

S18 Table P-values of Pair-wise T-tests from Mean Distance to Nearest Food Outlet of All Types, from Home, AY2013. (PDF)

S19 Table P-values of Pair-wise T-tests from Mean Distance to Nearest Food Outlet of All Types, from School, AY2013. (PDF)

S20 Table P-values of Pair-wise T-tests from Count of Food Outlets within 0.25 miles, from Home, AY2013. (PDF)

S21 Table P-values of Pair-wise T-tests from Count of Food Outlets within 0.25 miles, from School, AY2013. (PDF)
