# Supplementary material for: Disparities in food access around homes and schools for New York City children
Source: PLoS One. 2019 Jun 12;14(6):e0217341. doi: 10.1371/journal.pone.0217341 (PMC6561543; doi:10.1371/journal.pone.0217341)
Supplement: S1 Table — Sample includes NYC public school students in districts 1–32 with home and school address data and student-level demographic data. Students for whom a substantial proportion of their food environment lies outside of the city boundaries (those whose home or school is within half a mile from city borders) are excluded. For both home and school measurements, we conducted joint F-tests, which suggest significant differences (p<0.05), and pair-wise T-tests for multiple comparisons based on Bonferroni correction (28 pairs in total, with each pair tested on all four food outlets, separately). The majority of comparisons for home measurements were statistically significant (p<0.05) while for the school measurements, more often than not, the results indicated no statistically significant difference. S18 and S19 Tables present the T-test results (p-values), respectively. (PDF) [file pone.0217341.s001.pdf]

S1 Table. Mean nearest distance (in ft.) to food facilities from home and school, race and poverty interactions, AY2013

		Overall	Not low-income				Low-income			
		Total	White	Black	Hispanic	Asian	White	Black	Hispanic	Asian
Corner stores	Home	641	1131	759	691	809	984	601	463	679
		(665)	(1134)	(597)	(681)	(753)	(983)	(503)	(446)	(610)
	School	733	1097	786	745	826	1074	658	583	856
		(684)	(1096)	(666)	(699)	(740)	(957)	(527)	(503)	(705)
Fast-food outlets	Home	710	1000	836	723	792	955	726	573	718
		(599)	(935)	(600)	(622)	(683)	(817)	(520)	(451)	(563)
	School	744	929	802	726	781	999	724	627	844
		(584)	(793)	(605)	(584)	(633)	(758)	(510)	(473)	(625)
Wait-service outlets	Home	1095	1148	1427	969	952	1197	1444	908	926
		(916)	(1054)	(1036)	(824)	(783)	(994)	(1073)	(750)	(714)
	School	1084	1103	1268	971	1002	1252	1266	931	1094
		(827)	(947)	(979)	(765)	(781)	(875)	(959)	(689)	(738)
Any supermarkets	Home	1534	2166	1684	1604	1697	2087	1486	1296	1539
		(1163)	(1894)	(1091)	(1261)	(1239)	(1599)	(957)	(910)	(1022)
	School	1635	2181	1739	1632	1723	2271	1540	1384	1794
		(1220)	(2011)	(1166)	(1258)	(1188)	(1761)	(995)	(912)	(1126)
	N	789 520	55 600	15 349	22 989	26 104	68 274	190 525	304 231	106 447

Notes: Sample includes NYC public school students in districts 1-32 with home and school address data and student-level demographic data. Students for whom a substantial proportion of their food environment lies outside of the city boundaries (those whose home or school is within half a mile from city borders) are excluded. For both home and school measurements, we conducted joint F-tests, which suggest significant differences ($p<0.05$), and pair-wise T-tests for multiple comparisons based on Bonferroni

correction (28 pairs in total, with each pair tested on all four food outlets, separately). The majority of comparisons for home measurements were statistically significant ($p<0.05$) while for the school measurements, more often than not, the results indicated no statistically significant difference. S18 and S19 Tables present the T-test results (p-values), respectively.
