# Supplementary material for: Disparities in food access around homes and schools for New York City children
Source: PLoS One. 2019 Jun 12;14(6):e0217341. doi: 10.1371/journal.pone.0217341 (PMC6561543; doi:10.1371/journal.pone.0217341)
Supplement: S4 Table — Sample includes NYC public school students in districts 1–32 with home and school address data and student-level demographic data. Students for whom a substantial proportion of their food environment lies outside of the city boundaries (those whose home or school is within half a mile from city borders) are excluded. (PDF) [file pone.0217341.s004.pdf]

S4 Table. Mean count within 0.50 miles of food facilities from home and school, race and poverty interactions, AY2013

		Overall	Not low-income				Low-income			
		Total	White	Black	Hispanic	Asian	White	Black	Hispanic	Asian
Corner stores	Home	48.97	25.76	36.00	45.00	44.06	30.30	46.23	60.77	48.17
		(24)	(28)	(34)	(45)	(27)	(30)	(35)	(41)	(36)
	School	45.80	26.98	36.21	45.07	44.65	28.99	45.29	56.13	39.42
		(24)	(29)	(34)	(48)	(26)	(31)	(35)	(38)	(35)
Fast-food outlets	Home	57.15	64.03	45.01	62.82	72.18	41.15	46.38	62.63	64.29
		(85)	(46)	(63)	(88)	(50)	(35)	(43)	(68)	(53)
	School	66.47	70.14	58.35	70.77	75.99	47.44	61.48	73.15	64.83
		(84)	(68)	(73)	(91)	(61)	(68)	(72)	(76)	(73)
Wait-service outlets	Home	26.64	52.55	16.14	35.76	50.64	24.61	11.24	25.76	38.13
		(79)	(37)	(54)	(79)	(42)	(22)	(33)	(57)	(45)
	School	36.36	57.28	29.94	44.56	55.08	29.26	26.33	37.54	39.11
		(80)	(59)	(65)	(83)	(52)	(56)	(60)	(64)	(62)
Any supermarkets	Home	3.82	3.68	3.11	3.82	3.98	2.57	3.50	4.35	3.78
		(4)	(3)	(3)	(4)	(3)	(2)	(3)	(3)	(3)
	School	3.91	3.80	3.40	4.04	4.19	2.72	3.78	4.40	3.51
		(4)	(3)	(3)	(4)	(3)	(3)	(3)	(3)	(3)
	N	789 520	55 600	15 349	22 989	26 104	68 274	190 525	304 231	106 447

Notes: Sample includes NYC public school students in districts 1-32 with home and school address data and student-level demographic data. Students for whom a substantial proportion of their food environment lies outside of the city boundaries (those whose home or school is within half a mile from city borders) are excluded.
