# Supplementary material for: Disparities in food access around homes and schools for New York City children
Source: PLoS One. 2019 Jun 12;14(6):e0217341. doi: 10.1371/journal.pone.0217341 (PMC6561543; doi:10.1371/journal.pone.0217341)
Supplement: S6 Table — Sample includes NYC public school K-5 students in districts 1–32 with home and school address data and student-level demographic data. Students for whom a substantial proportion of their food environment lies outside of the city boundaries (those whose home or school is within half a mile from city borders) are excluded. (PDF) [file pone.0217341.s006.pdf]

S6 Table. Mean count within 0.25 miles of food facilities from home and school, race and poverty interactions, Grade K-5, AY2013

		Overall	Not low-income				Low-income			
		Total	White	Black	Hispanic	Asian	White	Black	Hispanic	Asian
Corner stores	Home	15.96	8.16	10.95	14.90	15.50	9.74	14.70	20.26	16.26
		(13)	(8)	(10)	(12)	(18)	(10)	(11)	(13)	(17)
	School	15.54	8.71	10.81	15.07	17.11	9.54	14.08	19.47	15.38
		(14)	(9)	(10)	(13)	(22)	(10)	(11)	(13)	(18)
Fast-food outlets	Home	18.06	19.91	13.28	19.92	23.25	12.61	13.78	19.89	19.94
		(19)	(27)	(16)	(22)	(31)	(17)	(12)	(16)	(24)
	School	18.23	20.47	13.47	20.98	24.15	13.14	13.62	20.25	18.85
		(20)	(25)	(16)	(23)	(33)	(17)	(14)	(18)	(25)
Wait-service outlets	Home	8.83	17.02	4.84	11.29	17.25	7.77	3.13	7.85	12.22
		(17)	(26)	(12)	(18)	(30)	(15)	(7)	(11)	(22)
	School	9.75	18.98	6.74	13.09	18.88	8.39	4.20	8.78	11.61
		(18)	(28)	(16)	(21)	(32)	(16)	(11)	(13)	(22)
Any supermarkets	Home	1.23	1.21	0.94	1.24	1.29	0.80	1.11	1.40	1.22
		(1)	(2)	(1)	(1)	(2)	(1)	(1)	(1)	(1)
	School	1.21	1.35	0.96	1.23	1.33	0.76	1.11	1.35	1.14
		(1)	(2)	(1)	(1)	(2)	(1)	(1)	(1)	(2)
	N	365 255	34 262	9 210	16 299	18 822	28 879	75 763	139 401	42 690

Notes: Sample includes NYC public school K-5 students in districts 1-32 with home and school address data and student-level demographic data. Students for whom a substantial proportion of their food environment lies outside of the city boundaries (those whose home or school is within half a mile from city borders) are excluded.
