# Supplementary material for: Disparities in food access around homes and schools for New York City children
Source: PLoS One. 2019 Jun 12;14(6):e0217341. doi: 10.1371/journal.pone.0217341 (PMC6561543; doi:10.1371/journal.pone.0217341)
Supplement: S11 Table — Sample includes NYC public school 6–8 grade students in districts 1–32 with home and school address data and student-level demographic data. Students for whom a substantial proportion of their food environment lies outside of the city boundaries (those whose home or school is within half a mile from city borders) are excluded. (PDF) [file pone.0217341.s011.pdf]

**S11 Table**. Mean count within 0.1 miles of food facilities from home and school, race and poverty interactions, Grade 6-8, AY2013

| | | Overall | Not low-income | | | | Low-income | | | |
|---|---|---|---|---|---|---|---|---|---|---|
| | | Total | White | Black | Hispanic | Asian | White | Black | Hispanic | Asian |
| Corner stores | Home | 2.00 | 1.15 | 1.65 | 1.64 | 2.14 | 1.78 | 2.05 | 1.78 | 3.05 |
| | | (4) | (2) | (3) | (3) | (4) | (3) | (3) | (3) | (5) |
| | School | 2.74 | 1.89 | 2.14 | 2.29 | 1.60 | 1.45 | 2.68 | 3.58 | 1.98 |
| | | (3) | (3) | (3) | (3) | (3) | (2) | (3) | (4) | (3) |
| Fast-food outlets | Home | 2.17 | 2.68 | 1.95 | 2.36 | 3.19 | 2.16 | 1.92 | 1.79 | 3.31 |
| | | (5) | (6) | (5) | (6) | (7) | (5) | (4) | (4) | (7) |
| | School | 3.51 | 4.70 | 3.82 | 4.04 | 3.37 | 2.37 | 3.14 | 3.90 | 3.23 |
| | | (5) | (7) | (6) | (6) | (6) | (5) | (5) | (5) | (6) |
| Wait-service outlets | Home | 1.06 | 2.14 | 0.80 | 1.57 | 2.48 | 1.28 | 0.35 | 0.84 | 2.09 |
| | | (4) | (6) | (3) | (5) | (7) | (4) | (2) | (3) | (6) |
| | School | 1.82 | 4.38 | 2.16 | 2.85 | 2.59 | 1.52 | 1.24 | 1.76 | 1.90 |
| | | (4) | (7) | (5) | (6) | (6) | (4) | (4) | (4) | (5) |
| Any supermarkets | Home | 0.13 | 0.15 | 0.12 | 0.13 | 0.17 | 0.13 | 0.14 | 0.11 | 0.18 |
| | | (0) | (0) | (0) | (0) | (1) | (0) | (0) | (0) | (1) |
| | School | 0.23 | 0.18 | 0.16 | 0.24 | 0.19 | 0.12 | 0.23 | 0.28 | 0.17 |
| | | (1) | (1) | (0) | (1) | (1) | (0) | (0) | (1) | (0) |
| | N | 176 770 | 10 386 | 2 661 | 3 350 | 3 878 | 16 326 | 45 223 | 69 727 | 25 219 |

**Notes:** Sample includes NYC public school 6-8 grade students in districts 1-32 with home and school address data and student-level demographic data. Students for whom a substantial proportion of their food environment lies outside of the city boundaries (those whose home or school is within half a mile from city borders) are excluded.
