# Supplementary material for: Disparities in food access around homes and schools for New York City children
Source: PLoS One. 2019 Jun 12;14(6):e0217341. doi: 10.1371/journal.pone.0217341 (PMC6561543; doi:10.1371/journal.pone.0217341)
Supplement: S14 Table — Sample includes NYC public school 9–12 grade students in districts 1–32 with home and school address data and student-level demographic data. Students for whom a substantial proportion of their food environment lies outside of the city boundaries (those whose home or school is within half a mile from city borders) are excluded. (PDF) [file pone.0217341.s014.pdf]

S14 Table. Mean count within 0.25 miles of food facilities from home and school, race and poverty interactions, Grade 9-12, AY2013

		Overall	Not low-income				Low-income			
		Total	White	Black	Hispanic	Asian	White	Black	Hispanic	Asian
Corner stores	Home	15.44	7.16	10.49	12.58	11.01	9.55	13.94	19.56	14.96
		(13)	(8)	(10)	(12)	(15)	(10)	(11)	(13)	(15)
	School	11.62	6.32	10.74	10.74	10.38	7.06	12.57	13.68	9.35
		(12)	(8)	(11)	(12)	(15)	(9)	(11)	(12)	(12)
Fast-food outlets	Home	16.66	17.40	12.18	17.03	18.17	12.43	13.27	19.41	18.54
		(18)	(27)	(15)	(21)	(28)	(17)	(12)	(16)	(23)
	School	22.06	17.54	21.79	23.61	29.49	13.46	22.36	25.63	18.36
		(29)	(30)	(29)	(32)	(49)	(23)	(27)	(31)	(28)
Wait-service outlets	Home	7.25	14.45	4.13	9.78	13.11	7.48	2.91	7.80	11.11
		(14)	(25)	(11)	(19)	(25)	(14)	(7)	(12)	(20)
	School	13.09	14.17	13.25	16.90	18.93	8.35	11.86	15.55	10.89
		(24)	(27)	(26)	(30)	(33)	(19)	(23)	(26)	(20)
Any supermarkets	Home	1.16	1.08	0.89	1.10	1.06	0.79	1.04	1.38	1.12
		(1)	(2)	(1)	(1)	(1)	(1)	(1)	(1)	(1)
	School	1.05	0.97	1.11	1.11	0.85	0.62	1.17	1.22	0.69
		(1)	(1)	(1)	(1)	(1)	(1)	(1)	(1)	(1)
	N	247 494	10 952	3 478	3 410	3 404	23 069	69 540	95 103	38 538

Notes: Sample includes NYC public school 9-12 grade students in districts 1-32 with home and school address data and student-level demographic data. Students for whom a substantial proportion of their food environment lies outside of the city boundaries (those whose home or school is within half a mile from city borders) are excluded.
