# Supplementary material for: Disparities in food access around homes and schools for New York City children
Source: PLoS One. 2019 Jun 12;14(6):e0217341. doi: 10.1371/journal.pone.0217341 (PMC6561543; doi:10.1371/journal.pone.0217341)
Supplement: S17 Table — (PDF) [file pone.0217341.s017.pdf]

S17 Table. Comparison of Mean and Median Measurements on Distance to Nearest Food Outlet and Count of Outlets within 0.25 Miles, AY2013.

		Distance to nearest		Count within 0.25 miles	
		Mean	Median	Mean	Median
Corner stores	Home	641	463	15.65	13
	School	733	532	13.79	12
Fast-food outlets	Home	710	573	17.31	13
	School	744	585	19.01	12
Wait-service outlets	Home	1095	902	8.02	3
	School	1084	907	10.43	3
Any supermarkets	Home	1534	1251	1.19	1
	School	1635	1243	1.13	1
	N	789 520	789 520	789 520	789 520
