# Supplementary material for: Disparities in food access around homes and schools for New York City children
Source: PLoS One. 2019 Jun 12;14(6):e0217341. doi: 10.1371/journal.pone.0217341 (PMC6561543; doi:10.1371/journal.pone.0217341)
Supplement: S18 Table — (PDF) [file pone.0217341.s018.pdf]

S18 Table. P-values of Pair-wise T-tests from Mean Distance to Nearest Food Outlet of All Types, from Home, AY2013

	Corner store	Fast food	Wait service	Supermarket
low-income Hispanic vs low-income Asian	0.000	0.000	0.000	0.000
low-income Black vs low-income Asian	0.000	0.005	0.000	0.000
low-income White vs low-income Asian	0.000	0.000	0.000	0.000
non-low-income Asian vs low-income Asian	0.000	0.000	0.001	0.000
non-low-income Hispanic vs low-income Asian	0.204	1.000	0.000	0.000
non-low-income Black vs low-income Asian	0.000	0.000	0.000	0.000
non-low-income White vs low-income Asian	0.000	0.000	0.000	0.000
low-income Black vs low-income Hispanic	0.000	0.000	0.000	0.000
low-income White vs low-income Hispanic	0.000	0.000	0.000	0.000
non-low-income Asian vs low-income Hispanic	0.000	0.000	0.000	0.000
non-low-income Hispanic vs low-income Hispanic	0.000	0.000	0.000	0.000
non-low-income Black vs low-income Hispanic	0.000	0.000	0.000	0.000
non-low-income White vs low-income Hispanic	0.000	0.000	0.000	0.000
low-income White vs low-income Black	0.000	0.000	0.000	0.000
non-low-income Asian vs low-income Black	0.000	0.000	0.000	0.000
non-low-income Hispanic vs low-income Black	0.000	1.000	0.000	0.000
non-low-income Black vs low-income Black	0.000	0.000	0.622	0.000
non-low-income White vs low-income Black	0.000	0.000	0.000	0.000
non-low-income Asian vs low-income White	0.000	0.000	0.000	0.000
non-low-income Hispanic vs low-income White	0.000	0.000	0.000	0.000
non-low-income Black vs low-income White	0.000	0.000	0.000	0.000
non-low-income White vs low-income White	0.000	0.000	0.000	0.000
non-low-income Hispanic vs non-low-income Asian	0.000	0.000	0.896	0.000
non-low-income Black vs non-low-income Asian	0.000	0.000	0.000	1.000
non-low-income White vs non-low-income Asian	0.000	0.000	0.000	0.000
non-low-income Black vs non-low-income Hispanic	0.000	0.000	0.000	0.000
non-low-income White vs non-low-income Hispanic	0.000	0.000	0.000	0.000
non-low-income White vs non-low-income Black	0.000	0.000	0.000	0.000
