# Supplementary material for: Disparities in food access around homes and schools for New York City children
Source: PLoS One. 2019 Jun 12;14(6):e0217341. doi: 10.1371/journal.pone.0217341 (PMC6561543; doi:10.1371/journal.pone.0217341)
Supplement: S19 Table — (PDF) [file pone.0217341.s019.pdf]

S19 Table. P-values of Pair-wise T-tests from Mean Distance to Nearest Food Outlet of All Types, from School, AY2013

	Corner store	Fast food	Wait service	Supermarket
low-income Hispanic vs low-income Asian	0.000	0.000	0.003	0.000
low-income Black vs low-income Asian	0.001	0.112	0.039	0.019
low-income White vs low-income Asian	0.016	0.037	0.157	0.099
non-low-income Asian vs low-income Asian	1.000	1.000	1.000	1.000
non-low-income Hispanic vs low-income Asian	0.366	0.063	0.224	0.720
non-low-income Black vs low-income Asian	1.000	1.000	0.134	1.000
non-low-income White vs low-income Asian	0.336	1.000	1.000	1.000
low-income Black vs low-income Hispanic	0.002	0.000	0.000	0.001
low-income White vs low-income Hispanic	0.000	0.000	0.000	0.000
non-low-income Asian vs low-income Hispanic	0.000	0.008	1.000	0.000
non-low-income Hispanic vs low-income Hispanic	0.000	0.000	1.000	0.000
non-low-income Black vs low-income Hispanic	0.000	0.000	0.000	0.000
non-low-income White vs low-income Hispanic	0.000	0.000	0.675	0.000
low-income White vs low-income Black	0.000	0.000	1.000	0.001
non-low-income Asian vs low-income Black	0.025	1.000	0.001	0.548
non-low-income Hispanic vs low-income Black	0.138	1.000	0.000	1.000
non-low-income Black vs low-income Black	0.001	0.026	1.000	0.001
non-low-income White vs low-income Black	0.000	0.034	1.000	0.022
non-low-income Asian vs low-income White	0.028	0.018	0.035	0.027
non-low-income Hispanic vs low-income White	0.000	0.000	0.000	0.000
non-low-income Black vs low-income White	0.009	0.053	1.000	0.068
non-low-income White vs low-income White	1.000	1.000	0.131	1.000
non-low-income Hispanic vs non-low-income Asian	1.000	1.000	1.000	1.000
non-low-income Black vs non-low-income Asian	1.000	1.000	0.001	1.000
non-low-income White vs non-low-income Asian	0.067	0.516	1.000	0.310
non-low-income Black vs non-low-income Hispanic	1.000	0.543	0.000	1.000
non-low-income White vs non-low-income Hispanic	0.000	0.002	1.000	0.015
non-low-income White vs non-low-income Black	0.038	1.000	1.000	0.594
