# Supplementary material for: Disparities in food access around homes and schools for New York City children
Source: PLoS One. 2019 Jun 12;14(6):e0217341. doi: 10.1371/journal.pone.0217341 (PMC6561543; doi:10.1371/journal.pone.0217341)
Supplement: S20 Table — (PDF) [file pone.0217341.s020.pdf]

S20 Table. P-values of Pair-wise T-tests from Count of Food Outlets within 0.25 miles, from Home, AY2013

	Corner store	Fast food	Wait service	Supermarket
low-income Hispanic vs low-income Asian	0.000	0.000	0.000	0.000
low-income Black vs low-income Asian	0.000	0.000	0.000	0.000
low-income White vs low-income Asian	0.000	0.000	0.000	0.000
non-low-income Asian vs low-income Asian	0.000	0.000	0.000	0.000
non-low-income Hispanic vs low-income Asian	0.000	1.000	0.000	0.001
non-low-income Black vs low-income Asian	0.000	0.000	0.000	0.000
non-low-income White vs low-income Asian	0.000	1.000	0.000	1.000
low-income Black vs low-income Hispanic	0.000	0.000	0.000	0.000
low-income White vs low-income Hispanic	0.000	0.000	0.002	0.000
non-low-income Asian vs low-income Hispanic	0.000	0.000	0.000	0.000
non-low-income Hispanic vs low-income Hispanic	0.000	0.001	0.000	0.000
non-low-income Black vs low-income Hispanic	0.000	0.000	0.000	0.000
non-low-income White vs low-income Hispanic	0.000	0.000	0.000	0.000
low-income White vs low-income Black	0.000	0.000	0.000	0.000
non-low-income Asian vs low-income Black	1.000	0.000	0.000	0.000
non-low-income Hispanic vs low-income Black	1.000	0.000	0.000	0.000
non-low-income Black vs low-income Black	0.000	0.020	0.000	0.000
non-low-income White vs low-income Black	0.000	0.000	0.000	0.000
non-low-income Asian vs low-income White	0.000	0.000	0.000	0.000
non-low-income Hispanic vs low-income White	0.000	0.000	0.000	0.000
non-low-income Black vs low-income White	0.000	0.082	0.000	0.000
non-low-income White vs low-income White	0.000	0.000	0.000	0.000
non-low-income Hispanic vs non-low-income Asian	1.000	0.000	0.000	0.115
non-low-income Black vs non-low-income Asian	0.000	0.000	0.000	0.000
non-low-income White vs non-low-income Asian	0.000	0.000	1.000	0.000
non-low-income Black vs non-low-income Hispanic	0.000	0.000	0.000	0.000
non-low-income White vs non-low-income Hispanic	0.000	1.000	0.000	0.001
non-low-income White vs non-low-income Black	0.000	0.000	0.000	0.000
