# Supplementary material for: Disparities in food access around homes and schools for New York City children
Source: PLoS One. 2019 Jun 12;14(6):e0217341. doi: 10.1371/journal.pone.0217341 (PMC6561543; doi:10.1371/journal.pone.0217341)
Supplement: S21 Table — (PDF) [file pone.0217341.s021.pdf]

S21 Table. P-values of Pair-wise T-tests from Count of Food Outlets within 0.25 miles, from School, AY2013

	Corner store	Fast food	Wait service	Supermarket
low-income Hispanic vs low-income Asian	0.000	0.105	1.000	0.000
low-income Black vs low-income Asian	1.000	1.000	0.020	0.588
low-income White vs low-income Asian	0.001	0.003	0.283	0.088
non-low-income Asian vs low-income Asian	1.000	1.000	0.175	1.000
non-low-income Hispanic vs low-income Asian	1.000	0.772	0.400	0.044
non-low-income Black vs low-income Asian	1.000	1.000	1.000	1.000
non-low-income White vs low-income Asian	0.001	1.000	0.017	0.699
low-income Black vs low-income Hispanic	0.000	0.000	0.000	0.000
low-income White vs low-income Hispanic	0.000	0.000	0.006	0.000
non-low-income Asian vs low-income Hispanic	1.000	1.000	0.741	1.000
non-low-income Hispanic vs low-income Hispanic	0.000	1.000	0.011	1.000
non-low-income Black vs low-income Hispanic	0.000	0.000	0.041	0.000
non-low-income White vs low-income Hispanic	0.000	1.000	0.009	1.000
low-income White vs low-income Black	0.000	0.000	1.000	0.000
non-low-income Asian vs low-income Black	1.000	1.000	0.024	1.000
non-low-income Hispanic vs low-income Black	1.000	0.017	0.000	1.000
non-low-income Black vs low-income Black	0.000	1.000	0.385	0.396
non-low-income White vs low-income Black	0.000	1.000	0.000	1.000
non-low-income Asian vs low-income White	0.099	0.051	0.067	0.074
non-low-income Hispanic vs low-income White	0.000	0.000	0.000	0.000
non-low-income Black vs low-income White	0.002	0.046	1.000	0.001
non-low-income White vs low-income White	1.000	0.000	0.000	0.000
non-low-income Hispanic vs non-low-income Asian	1.000	1.000	1.000	1.000
non-low-income Black vs non-low-income Asian	1.000	0.580	0.087	1.000
non-low-income White vs non-low-income Asian	0.063	1.000	1.000	1.000
non-low-income Black vs non-low-income Hispanic	0.000	0.000	0.000	0.040
non-low-income White vs non-low-income Hispanic	0.000	1.000	0.237	1.000
non-low-income White vs non-low-income Black	0.000	0.468	0.000	1.000
